# Diagnostic therapeutic care pathway for pediatric food allergies and intolerances in Italy: a joint position paper by the Italian Society for Pediatric Gastroenterology Hepatology and Nutrition (SIGENP) and the Italian Society for Pediatric Allergy and Immunology (SIAIP)

**DOI:** 10.1186/s13052-022-01277-8

**Published:** 2022-06-10

**Authors:** Roberto Berni Canani, Carlo Caffarelli, Mauro Calvani, Alberto Martelli, Laura Carucci, Tommaso Cozzolino, Patrizia Alvisi, Carlo Agostoni, Paolo Lionetti, Gian Luigi Marseglia

**Affiliations:** 1 Department of Translational Medical Science, University of Naples Federico II, Naples, Italy; 2 CEINGE-Biotecnologie Avanzate s.c.ar.l. University of Naples Federico II, Naples, Italy; 3 European Laboratory for the Investigation of Food-Induced Diseases, University of Naples, Federico II, Naples, Italy; 4 Task Force for Microbiome Studies, University of Naples Federico II, Naples, Italy; 5 Pediatric Clinic, Department of Medicine and Surgery, University of Parma, Parma, Italy; 6 Pediatric Unit, S. Camillo Forlanini Hospital, Rome, Italy; 7 Department of Pediatrics, G. Salvini Hospital, Garbagnate Milanese, Milan, Italy; 8 Pediatric Gastroenterology Unit, Maggiore Hospital, Bologna, Italy; 9 Department of Clinical Sciences and Community Health, University of Milan, Milan, Italy; 10 Pediatric Intermediate Care Unit, Fondazione IRCCS Ca’ Granda Ospedale Maggiore Policlinico, Milan, Italy; 11 Pediatric Gastroenterology and Nutrition Unit, Meyer Children’s Hospital, Florence, Italy; 12 Pediatric Clinic, IRCCS “S. Matteo” Foundation, University of Pavia, Pavia, Italy

**Keywords:** Adverse food reactions, Food allergy, Carbohydrates intolerance, Lactose intolerance, Anaphylaxis, Component resolved diagnosis, Oral food challenge

## Abstract

Epidemiologic data suggest an increased prevalence of pediatric food allergies and intolerances (FAIs) during the last decades. This changing scenario has led to an increase in the overall healthcare costs, due to a growing demand for diagnostic and treatment services. There is the need to establish Evidence-based practices for diagnostic and therapeutic intervention that could  be adopted in the context of public health policies for FAIs are needed.

This joint position paper has been prepared by a group of experts in pediatric gastroenterology, allergy and nutrition from the Italian Society for Pediatric Gastroenterology Hepatology and Nutrition (SIGENP) and the Italian Society for Pediatric Allergy and Immunology (SIAIP). The paper is focused on the Diagnostic Therapeutic Care Pathway (DTCP) for pediatric FAIs in Italy.

## Introduction

**Fig. 1:**
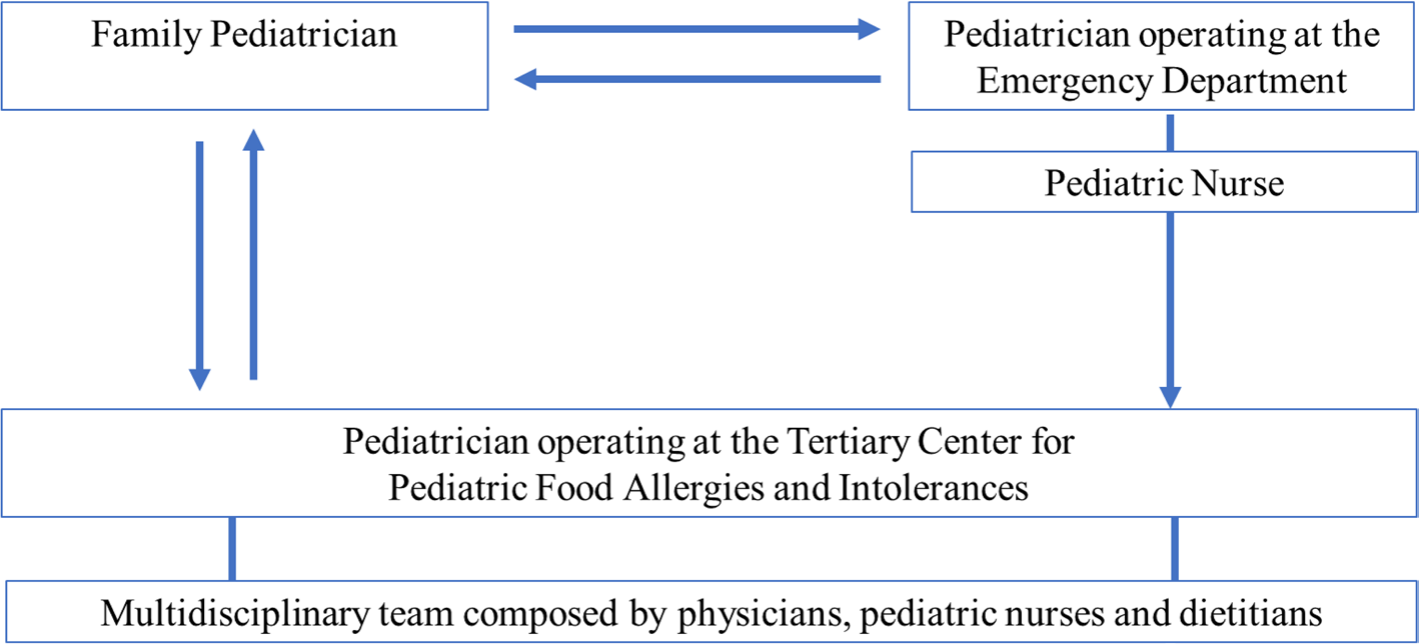
Toward an integrated approach to pediatric patients with Food Allergies and Intolerances. The healthcare professionals approaching pediatric patients with FAIs cooperate in a circular continuum in the management of these patients. Based on the symptoms severity, patients could be referring to the ED or to FP. The physician operating at the ED, after full stabilization of the symptoms, can refer the patient to the FP or to the tertiary center for the diagnosis and the management of  FAIs (protected outpatient pathway). If the patient primarily refers to the FP, he should extensively evaluate the anamnestic and clinical features of the patient, treat any symptom or referring to the ED in case of acute symptoms that rapidly involve multiple organs. In case of a suggestive history of FAIs, the FP should request a specialist evaluation by the tertiary center for pediatric FAsI. Abbreviations: FAIs, Food Allergies and Intolerances. ED, Emergency Department. FP, Family Pediatrician

This joint position paper has been prepared by a group of experts in pediatric gastroenterology, allergy and nutrition from the Italian Society for Pediatric Gastroenterology Hepatology and Nutrition (SIGENP) and the Italian Society for Pediatric Allergy and Immunology (SIAIP). The paper is focused on the Diagnostic Therapeutic Care Pathway (DTCP) for pediatric food allergies ad intolerances (FAIs) in Italy. The group considered this a priority topic to support clinical practice of healthcare professionals approaching pediatric subjects affected by these conditions. All the activities and procedures, that are considered as a minimum essential level, have been included in a circular continuum of activities provided by different healthcare professionals **(**Fig. 1**)**. The need to elaborate this document derives from the significant increase in the prevalence of FAIs in the last two decades which has led to a growing demand for diagnostic and therapeutic services, which are often incongruous and inappropriate (such as the use of non-scientifically validated diagnostic tests and “self-therapy”) with a consequent increase in the overall healthcare costs and diagnostic errors and delays [1–3].

The design of this DTCP is inspired by the logic of governance of activities in which each health care professional figure plays a defined role in following established practices to guarantee the optimal management of the patients throughout the national territory.

### Objectives

The main objectives of the DTCP are:

1. To define the correct criteria to identify subjects with FAIs;
2. To define the appropriate procedures for the diagnosis, treatment, and follow-up of these conditions;
3. To define the skills, roles and responsibilities of the health care professionals involved in the process of “care” of FAIs to reduce delays and diagnostic errors, health care costs, and risks for children affected by FAIs.

### Definitions

Based on a consensus of the European Academy of Allergy and Clinical Immunology (EAACI), subsequently revisited by the American Gastroenterology Association (AGA), adverse food reactions can be classified into toxic and non-toxic [4, 5], toxic food reactions affect every individual and are dependent on the amount of food ingested contaminated by toxic substances that can be contained in food naturally or may be produced following its handling (e.g., mushroom poisoning, gastroenteritis from bacterial toxins contained in spoiled foods).

The DTCP focuses on non-toxic food adverse reactions, linked to individual susceptibility to certain foods, which from a pathogenetic point of view are divided into:

- food allergies (FA) (immune-mediated).
- food intolerances (FI) (not immune-mediated).

Food allergies can in turn be divided mainly into two categories: IgE-mediated forms, attributable to an initial sensitization process to certain proteins against which the immune system develops IgE class antibodies responsible for acute symptoms onset (usually within 2 hours of food intake); and non-IgE-mediated forms, characterized by the involvement of humoral and/or cellular components with a later onset (few hours to few days after food intake) and with pathophysiological mechanisms not yet fully defined. In some cases the clinical picture may be attributable to a mixed IgE- and non-IgE-mediated mechanism (mixed forms) [6, 7].

Food intolerances are non-immune adverse reactions to food. These reactions can be attributable to an enzymatic defect (e.g., lactose intolerance) or to alteration of transport mechanisms (e.g., fructose intolerance) or to other mechanisms (intolerance to Oligo-, Di-, Monosaccharides and Fermentable Polyols, FODMAPs; secondary reactions to ingestion of vasoactive amines or additives contained in food) [8].

### Epidemiology

FAIs are among the most common chronic conditions in the pediatric age and are recognized as a global health problem. Epidemiology of FA has changed during the last two decades with a dramatic increase in the prevalence, severity of clinical manifestations, leading to an increase in hospital admissions, medical visits, treatments, burden of care on families and economic impact, with significant direct costs for the families and the healthcare system [5, 9, 10].

According to Italian National Institute of Statistics (ISTAT) data obtained from sample surveys updated to 2016, subjects suffering from allergic diseases in Italy showed an increasing trend from 9.8% in 2010 to 10.7% in 2016, preferentially involving subjects up to 18 years of age. In Italy during the last 20 years there has been an increase of over 400% in the number of visits to the Emergency Department (ED) due to food-induced anaphylaxis [9]. Furthermore, according to the data released by the Italian Ministry of Health updated to 2017, in Italy the subjects suffering from FA equal 1,800,000 and it is estimated that about 50% are affected by non-IgE-mediated forms in the pediatric age [10], while lactase deficiency affects an average of 40–50% of the population. This progressive increase reflects the European and global situation. Cow’s milk allergy (CMA) is one of the most frequent FA in children with a prevalence ranging from 2 to 6% in Europe [9]. In Italy, according to the Italian National Institute of Statistics (ISTAT), the CMA estimated costs are about 20 million euros per year, to which about 52 million euros should be added, deriving from the use of special formulas and cow’s milk protein-free foods [11].

Over the years there has also been an increase in the prevalence of “new” clinical manifestations of FAIs of pediatric gastroenterology interest (such as eosinophilic disorders of the gastrointestinal tract or Food Protein-Induced Enterocolitis Syndrome FPIES) that require a multidisciplinary and complex diagnostic-therapeutic planning [12].

### Symptoms

The signs and symptoms that could raise the suspicion of FA or FI are multiple. They depend on the type of pathogenetic mechanism (FI vs IgE- or non-IgE-mediated or mixed forms of FA), involved food, quantity ingested, food preparation method (thermolabile and thermostable allergens), type of contained allergens, exposure mode (concomitant intake of drugs or physical exercise), specific factors of the host (age, eating habits, degree and type of sensitization, presence of other allergic diseases) and presence of any concomitant comorbidities (disorders that cause intestinal mucosa damage are to be considered conditions favoring sensitization and allergic reaction). Some clinical pictures (anaphylaxis, angioedema, asthma, urticaria), particularly when arise acutely (generally within 2 hours) after food contact or ingestion, strongly evoke the suspect of IgE-mediated FA [13]. An exception could be given by FPIES, a non-IgE-mediated FA characterized by an acute clinical picture (repeated vomiting that occurs within 1–4 hours after ingestion of the suspected food accompanied by other symptoms such as lethargy, pallor, hypotension, diarrhea and hypothermia) in the absence of skin or respiratory symptoms typical of IgE-FA (Table 1**) (*enter*** Table 1***here***) [14, 15]. Other clinical manifestations, if chronic or subacute and related to the gastrointestinal system, are not pathognomonic and can be symptoms of non-IgE-mediated FA, FI or other chronic conditions (functional gastrointestinal disorders, inflammatory bowel diseases, neoplasms, chronic infections). Thus, in some cases, the suspicion of FA or FI must be placed after excluding other possible diseases in the differential diagnosis.

**Table 1:** Diagnostic criteria for Food Protein-Induced Enterocolitis Syndrome (FPIES)

**Acute FPIES Diagnostic Criteria** The diagnosis of FPIES requires that the patient meet the major criterion and at least 3 minor criteria. If only a single episode has occurred, a diagnostic OFC should be strongly considered to confirm the diagnosis, especially because viral gastroenteritis is so common in this age group. Furthermore, although not a criterion for diagnosis, it is important to recognize that acute FPIES reactions will typically completely resolve over a matter of hours compared with the usual several-day time course of gastroenteritis. The patient should be asymptomatic and growing normally when the offending food is eliminated from the diet. Major criterion: Vomiting in the 1- to 4 hours after ingestion of the suspect food and the absence of classic IgE-mediated allergic skin or respiratory symptoms. Minor criteria: 1.A second (or more) episode of repetitive vomiting after eating the same suspect food 2.Repetitive vomiting episode after 1–4 hours after eating a different food 3.Extreme lethargy with any suspected reaction 4.Marked pallor with any suspected reaction 5.Need for Emergency Departed visit with any suspected reaction 6.Need for intravenous fluid administration with any suspected reaction 7.Diarrhea within 24 hours (usually 5–10 hours) 8.Hypotension 9.Hypothermia	
**Chronic FPIES Diagnostic Criteria** Severe presentation: when the offending food is ingested on a regular basis (e.g., infant formula); intermittent but progressive vomiting and diarrhea (occasionally with blood) develop, sometimes with dehydration and metabolic acidosis. Milder presentation: lower doses of the problem food (e.g., solids food or food allergens in breast milk) lead to intermittent vomiting and/or diarrhea, usually with poor weight gain/failure to thrive but without dehydration or metabolic acidosis. The most important criterion for chronic FPIES diagnosis is resolution of the symptoms within days after elimination of the offending food(s) and acute recurrence of symptoms when the food is reintroduced, onset of vomiting in 1–4 hours, diarrhea within 24 hours (usually 5–10 hours). Without confirmatory OFC, the diagnosis of chronic FPIES remains presumptive.	
**Diagnostic criteria for the interpretation of OFCs** Major criterion: Vomiting in the 1- to 4 hours period after ingestion of the suspect foods and the absence of classic IgE-mediated allergic skin or respiratory symptoms. Minor criteria: 1.Lethargy 2.Pallor 3.Diarrhea within 5–10 hours after food ingestion 4.Hypotension 5.Hypothermia 6.Increased neutrophil count of ≥1500 neutrophils above the baseline count.	

The OFC is considered diagnostic of FPIES, i.e., positive, if the major criterion is met with at least 2 minor criteria. However, two important remarks need to be considered: (1) With the rapid use of ondansetron, many of the minor criteria, such as repetitive vomiting, pallor, and lethargy may be averted; and (2) Not all facilities performing challenges have the ability to perform neutrophil counts in a timely manner

Abbreviations: *OFC* Oral Food Challenge, *FPIES* Food Protein-Induced Enterocolitis Syndrome

Toxic forms include scombroid syndrome, in which inadequately preserved fish contains large amounts of histamine, derived from the bacterial metabolism of the amino acid L-histidine in the fish muscle, which may cause urticarial rash, systemic symptoms (headache, tachycardia, hypotension) and gastrointestinal symptoms including nausea, vomiting, diarrhea, abdominal pain [8].

Finally, among the pharmacological forms, which occur because the ingested food contains substances with pharmacological-like activity, may determine the onset of gastrointestinal symptoms. An example is intoxication by glycoalkaloids (including α-solanine) contained in potatoes. In particular conditions or in the presence of excessive ripening, the accumulation of these substances can cause a clinical picture with vomiting, severe diarrhea and abdominal pain and other systemic symptoms, due to the inhibition of acetylcholinesterase [8].

In Table 2 are depicted the main symptoms and clinical entities of pediatric FA and in Table 3 the main clinical features of different forms of Non-IgE-mediated FA ***(enter*** Table 2***and*** Table 3***here)***.

**Table 2:** Symptoms and clinical entities of FA in the pediatric age

Symptoms/signs	IgE-mediated FA	Mixed form (IgE/non-IgE-mediated FA)	Non-IgE-mediated FA
**Gastrointestinal**	- Nausea/vomiting - Diarrhea - Abdominal pain - Itching of the oral cavity - Tongue edema	- Nausea/vomiting - Sialorrhea - Diarrhea - Colic - Constipation - Abdominal pain - Dysphagia - Dyspepsia - Retrosternal pyrosis - Loss of appetite - Hematochezia/ melaena - Malabsorption - Poor growth/weight loss - Food impaction	- Nausea/vomiting - Sialorrhea - Diarrhea - Colic - Constipation - Abdominal pain - Dysphagia - Dyspepsia - Retrosternal pyrosis - Loss of appetite - Hematochezia/ melaena - Malabsorption - Poor growth/weight loss
**Respiratory**	- Itchy nose/nasal congestion - Rhinorrhea - Sneezing - Wheezing/coughing/ dyspnea - Laryngeal stridor - Thoracic constriction - Conjunctival tearing, itching and hyperemia		- Interstitial lung disease
**Cutaneous**	- Wheals - Edema of the subcutaneous tissues - Rapid onset erythema or rash - Pruritus	- Eczematous lesions	
**Others**	- Hypotension - Pallor - Lethargy - Shock		- Hypotension - Pallor - Lethargy - Shock
**Clinical entities**	**IgE-mediated FA**	**Mixed form** **(IgE/non-IgE-mediated FA)**	**Non-IgE-mediated FA**
	- Anaphylaxis - Food-dependent exercise- induced anaphylaxis - Oral allergy syndrome - Acute repetitive vomiting and/or abdominal pain and/or diarrhea - Asthma and oculorhinitis - Urticaria and angioedema	- Atopic dermatitis - Eosinophilic disorders of the gastrointestinal tract	- Food Protein-Induced Enterocolitis Syndrome (FPIES) - Food Protein-Induced Allergic Proctocolitis (FPIAP) - Food Protein- induced Enteropathy (FPE) - Food induced motility disorders (FPIMD) (constipation, colic, gastroesophageal reflux disease) - Heiner Syndrome

Abbreviation: *FA* Food allergy

**Table 3:** Main clinical features of Non-IgE-mediated FA in the pediatric age

Non-IgE-mediated FA	Main clinical features
Food Protein-Induced Enterocolitis Syndrome (FPIES)	**Cardinal symptoms:** Acute FPIES: Vomiting 1–4 h after ingestion Chronic FPIES: intermittent but progressive vomiting and diarrhoe **Additional symptoms:** Acute FPIES: pallor, lethargy, hypovolaemia, hypotension, diarrhoea Chronic FPIES: faltering growth
Food Protein-Induced Allergic Proctocolitis (FPIAP)	**Cardinal symptoms:** Blood in stool **Additional symptoms:** Occasional loose stools, mucous in the stools, painful flatus, anal excoriation
Food Protein- induced Enteropathy (FPE)	**Cardinal symptoms:** Diarrhoea, failure to thrive **Additional symptoms:** Mucus and bloating, intermitting vomiting, abdominal pain, faltering growth, hypoalbuminemia
Food induced motility disorders (FPIMD) -Constipation -Colic -Gastroesophageal reflux disease (GORD)	Persistent FPIMD symptoms often coexisting, associated with atopic dermatitis and not responsive to conventional treatment **Cardinal symptoms:** Straining with soft stools **Additional symptoms:** Faecal impaction, bloating, abdominal pain **Cardinal symptoms:** Colic based on Rome IV consensus [16] **Additional symptoms:** Abnormal stool patterns, faltering growth **Cardinal symptoms:** Intermitted painful vomiting/regurgitation **Additional symptoms:** Faltering growth, feeding difficulties backarching with pain

Abbreviation: *FA* Food allergy

### Diagnostic approach for pediatric food allergy

The initial approach by healthcare professionals operating at ED and/or by the Family Pediatrician (FP) could be relevant for the evaluation of potential indication for a specialist consultation. The ED physician and/or FP should perform an anamnestic evaluation of the patient concerning [17]:

- main clinical features.
- recurrence of the clinical manifestations.
- time between food intake and symptoms onset.
- duration of symptoms.
- mode of symptom resolution (spontaneously or after therapy).
- type, quantity, and cooking method of foods taken in the 24 hours before clinical picture onset.
- concomitant intake of drugs (painkillers, antibiotics, etc.)
- relationship with other conditions (gastrointestinal system, etc.)
- relationship with physical activity after a meal.
- presence of similar symptoms in other diners.
- personal or family medical history for FA.

If the anamnestic evaluation leads to a suspected diagnosis of FAI, the ED physician and/or the FP should ask for a visit to a tertiary center.

#### First level tests

Skin allergy tests (skin prick tests, SPT) are allergy screening tests commonly used to identify the presence of specific IgE for food allergens and are performed by placing a drop of the allergen extract on the skin (usually the volar portion of the forearm) and then pricking the skin, through the drop, with a metal lancet with a 1-mm disposable tip. The tests are then evaluated 15 minutes after application; wheals at least 3 mm in diameter larger than the negative control are considered positive [18, 19].

Food allergens are composed by several molecules, some stable to heat, storage and digestion and others less stable in which the allergenicity could be significantly reduced when the food is exposed to high temperature [20]. The allergenic extracts for SPT containing proteins stable to heat and gastric digestion such as casein from cow’s milk, ovomucoid from egg etc., have a high negative predictive value [21]. Allergenic extracts for SPT of other foods, such as vegetables, have a low negative predictive value as they contain thermolabile molecules such as profilins. For these allergens it may be useful to use “prick by prick” technique with fresh food [22]. In the case of suggestive symptoms of FA and positive SPT, the diagnosis is certain if the clinical picture is compatible with anaphylaxis, while it is only probable in all other cases. To obtain a definitive diagnosis it is necessary to perform the oral food challenge (OFC) after a diagnostic elimination diet. On the other hand, if the skin test results are doubtful or negative in contradiction with the clinical history, it is possible to proceed with the specific serum IgE assay and with an elimination diet followed by diagnostic OFC (second level diagnosis) [23].

#### Second level tests

The measurement of total and food-specific serum IgE are considered second level tests. The determination of total serum IgE is not helpful for the diagnosis of FA. On the contrary, the evaluation of food-specific serum IgE could be helpful in the diagnostic approach when the SPT are doubtful or negative in contradiction with the clinical history, or when it is not possible to perform the SPT (inability to suspend the antihistamine or steroid treatment, presence of skin lesions, dermographism).

#### Third level tests

The assay of IgE antibodies against the food individual allergenic molecules also called Component-resolved diagnostic (CRD) allows to establish the sensitization profile of each patient, to establish whether it is primary and/or secondary food allergens sensitization to cross-reactive molecules (panallergens). CRD has prognostic value and can potentially contribute to the identification of reaction severity [24, 25]. Furthermore, CRD approach could be helpful in subjects with specific serum IgE false positivity. This could be the case of polyclonal IgE activation with high total IgE serum levels. It may be also due to specific IgE against cross-reactive carbohydrate determinants (anti-CCD IgE) that do not cause clinical symptoms but can determine an increase of specific IgE serum levels for foods or environmental allergens [26].

Therefore, the CRD approach has various implications in clinical practice, especially when a sensitization to multiple foods or between foods and inhalant allergens occurs. These processes are characterized by cross-reactivity between allergenic molecules with high levels of homology, expressed by different foods and/or inhalant allergens such as plant foods and pollen or crustaceans and mites, also called panallergens. The main families of panallergens are responsible for the so-called “pollen-food syndrome” and are the following:

- Profilins are molecules that are generally inactivated by heat and proteolysis. They are also called Bet-v2 homologous, the birch profilin. They are usually responsible for oral allergic syndrome (itching of the lips, tongue, palate, ears and throat and/or angioedema of the same sites) induced by ingestion of raw foods, Usually, the primary sensitization is towards grasses or birch; this family includes Mal-d4 of apple, Pru-p4 of peach, Heb-v8 of latex [27]. An exception is the celery profilin (Api g 4), which is heat resistant and can consequently cause symptoms even with cooked food.
- Pathogenesis Related Proteins-10 (PR-10) are molecules partially inactivated by heat and proteolysis, usually responsible for oral allergic syndrome, or for non-serious systemic reactions. They are also defined Bet-v1homologous, and the primary sensitization is generally towards the Bet-v1 (PR-10 of the birch); this family includes e.g., Pru-av1 of the cherry, and Pru-ar1 of the apricot. The only PR-10 known to cause systemic reactions is Gly-m4 from soy [28].
- Lipid Transfer Proteins (LTP) are molecules stable to heat and proteolysis, responsible for systemic reactions, also defined as Pru-p3 homologous, the LTP of peach which contains almost all the epitopes of the LTP present in nature. This family includes Mal-d3 of apple, Art-v3 of mugwort, and typically they are found in the peel of rosaceae, almonds and in the edible seeds of kiwi and tomatoes [29].
- Seed storage proteins are allergens resistant to heat and digestion, are associated with a high risk of serious reactions and are present in legumes, nuts, and seeds, with partial cross-reactivity between the different species. This family includes Ara-h1, 2 and 3 of the peanuts, Cor-a 9, 11 and 14 of the hazelnuts, Jug-r 1, 2 and 4 of the walnut, Gly-m 5 and 6 of the soy [30].
- Serum albumin are cross-reactive proteins present in mammals, responsible for allergic reactions, even systemic, to meat (e.g., “Cat-pork syndrome” for homology between Fel-d2 from cats and serum albumin from pigs) [31] and to milk (e.g., “Milk-beef syndrome” for homology between Bos-d6 of milk and cow) [32].
- Parvalbumins are the major allergens of fish with bones. They are heat- and digestive-resistent proteins responsible for severe systemic reactions. Sensitization generally occurs by ingestion but can also occur by contact or inhalation of proteins in aerosols, generated during cooking or food processing; examples of parvalbumins are Gad-c1 from cod, and Cyp-c1 from carp [33].
- Tropomyosins are the major allergens of crustaceans. They are heat- and digestive-resistant proteins and are associated with the risk of severe allergic reactions. They have a high cross-reactivity in Phylum Arthropods. The main members of this family are Der p 10 of the dust mite, and Pen-a1 of the shrimp [34].
- Galactose-alpha-1,3-galactose (alpha-Gal) is the allergen responsible for allergic reactions, including anaphylaxis, with an IgE-mediated mechanism, but with delayed onset (after 2–8 hours) after ingestion of red meat or jellies. It is an oligosaccharide resistant to industrial treatments; being slowly absorbed with the lipids of red meat, it reaches the bloodstream through the thoracic duct, thus triggering delayed reactions, even severe ones [35].
- Gibberellin-regulated protein (GRP) has been identified as trigger allergen in patients with peach allergy (Pru p 7) and cypress sensitization containing a GRP homologous (Cup s 7). The clinical picture could vary from Oral allergic syndrome (OAS) to anaphylaxis. GRP is contained in many fruits such as orange (Ct s 7), Japanese apricot (Pru m 7), pomegranate (Pum g 7), kiwi [36].
- Most egg allergens are found in egg white and are Gal d 1 (ovomucoid, being the main allergen, a thermo- and pepsin-resistant protein, marker of a possible severe allergic reaction [37]. Persistent specific IgE towards this component are associated with a greater risk of allergy persistence in adulthood and of subsequent sensitization to inhalants); Gal d 2 (ovalbumin, partially thermostable, well digested at very low pH) [38]; Gal d 3, (ovotransferrin, thermolabile, partially cross-reacting with chicken serum albumin) [39]; Gal d 4, (lysozyme, thermolabile on cooking over 80 °C for at least 2 minutes, often hidden allergen because used as additive for its bacteriostatic actions) [40]. The main specific allergens of the yolk are Gal d 5 (livetin, with possible cross-reactivity with livetin of the chicken) [41] and Gal d 6 (detected in many patients with yolk allergy) [42].
- The main allergens of cow’s milk are Bos d 4 (alpha-lactalbumin) [43], Bos d 5 (beta-lactoglobulin), Bos d 6 (whey albumin). They are all thermolabile proteins, therefore many food allergic children could tolerate these antigens in cooked food. Bos d 6 represents the major allergen of beef and is thus associated with the risk of adverse reactions to raw red meat. Bos-d8 (casein), is a thermostable protein, potentially related to severe clinical reactions; represents a potential hidden allergen because it is used as an additive in the food industry; it also has a high homology with sheep and goat caseins [44].

Each food may therefore contain allergenic molecules belonging to different families and, according to the sensitization profile, it can induce reactions from mild to severe such as anaphylactic shock. For example, the allergens Pru-p1 (PR-10), Pru-p3 (LTP), Pru-p4 (profilins) are present in peaches [45]. Therefore, a patient with peach allergy, may show very different clinical reactions: from mild reactions such as oral itching, typical of sensitization towards profilins, to more severe reactions up to anaphylactic shock, possible in the case of LTP sensitization. On the other hand, even if panallergens, such as LTP contained in different foods, have important structural homologies, this does not necessarily imply that cross-sensitization leads to clinical cross-reactivity.

CRD can be performed through the quantitative dosage of the single molecules that may be available (singleplex) or through pre-packaged panels through microarrays, more frequently semi-quantitative tests [46]. The multiplex tests allow to detect specific reactivity towards numerous allergenic molecules from inhalants to foods. These tests can be useful in patients with polysensitization allowing the identification of the allergens molecular profile, or in subjects with idiopathic anaphylaxis, where it is not possible to identify the causative food or when the molecule is not available for individual measurement.

The costs, the need for continuous updating on the list of available molecules, their characteristics and the results interpretation place molecular diagnostics in a strictly specialized field.

The Atopy Patch Test (APT) is a simple, safe, and low-cost test, potentially useful in the diagnostic approach to children with suspected non-IgE mediated FA. It is carried out by applying a drop of fresh food (equal to 50 μl) (e.g., whole cow’s milk, eggs, powdered wheat dissolved in water or saline: 1 g /10 ml) using a hypoallergenic patch containing a 12 mm aluminum well on which to lay an allergen absorbent cellulose disc [47]. Double-distilled water is preferable as a negative control. The test must always be interpreted as part of a careful evaluation of clinical history, response to the elimination diet and the result of the OFC. However, due to the lack of standardization and the variability of the results reported in the literature, there is no consensus on the possibility of using alone in the FA diagnosis [48].

Oral food challenge represents the “gold standard” for the diagnosis of FA, both for the IgE- and non-IgE-mediated form. The OFC should be performed in all cases of suspected FA. Exceptions are patients with suggestive symptoms of FPIES (Table 1) after the intake of a single food and patients with clinical symptoms typical of an IgE-mediated and severe FA (anaphylaxis) arising after the intake of only one type of food, resulted positive at SPT [49]. In these cases, it is preferable to postpone the OFC to avoid the onset of severe reactions [17]. In all cases, before the OFC it is necessary to explain both to the child and the parents the cost/benefit ratio of the test, and to acquire the written informed consent. For this reason, the OFC must be performed in a hospital setting with confirmed experience with this procedure and with emergency medication and resuscitation equipment readily available [50]. In the clinical practice, the OFC is commonly performed in open fashion. It constitutes the simplest way to perform the OFC, because both the medical doctor and the patient (and family members) are aware of which food is administered. Open OFC is reliable mostly when the patient aged less than 3 years and an immediate reaction with objective symptoms is expected. In some situation to avoid that the emotional component affects the genesis and/or evaluation of symptoms, blinded tests are used. Blinded OFC can be divided in single blind (only the medical doctor knows the food administered) or double-blind (neither the medical doctor nor the patient are aware of the food administered) in which the allergenic food is administered mixed with other foods, so as not to be recognized. The food is administered gradually at increasing doses every 15–30 minutes, depending on the protocol, until the maximum dose that generally correspond to the usual daily ration of that food. Exception is FPIES in which it has been proposed to administer the food total dose divided in 3 equal parts, at intervals of 30 minutes. Usually, the total dose to be administered should be calculated by multiplying 0.15–0.3 g of the suspected allergenic protein per kg of the patient’s body weight. The maximum dose should not exceed 3 g total of food protein or/and 100 ml for liquids. The OFC is stopped at the onset of the first objective or even subjective symptoms if repeated, to stop the allergic reaction [23]. The OFC is considered positive when clear objective symptoms of a possible allergic reaction arise or if severe and persistent subjective symptoms (e.g., abdominal pain) occur and are repeated at least 3 times. In the case of FPIES, the OFC is considered positive if, in addition to the major criterion, at least 2 minor criteria occur **(**Table 1**).** In some cases, the physician could consider the OFC positive if just a major criterion occurs. The OFC is considered negative if no symptoms occur within 2 hours of the food full dose assumption in the IgE-mediated forms, and within 6 hours in the acute FPIES. It should be noted that the symptoms of non-IgE-mediated forms may occurs even after a few days from the start of the food administration; for this reason, the OFC is considered negative if no symptoms occur in the following 7–10 days, by administering the suspect food at home. Finally, the OFC is defined as inconclusive (or conclusive only for partial tolerance) if it is suspended before the assumption of the food total dose and no symptoms occurred.

##### Gastroenterological tests

**Fig. 2:**
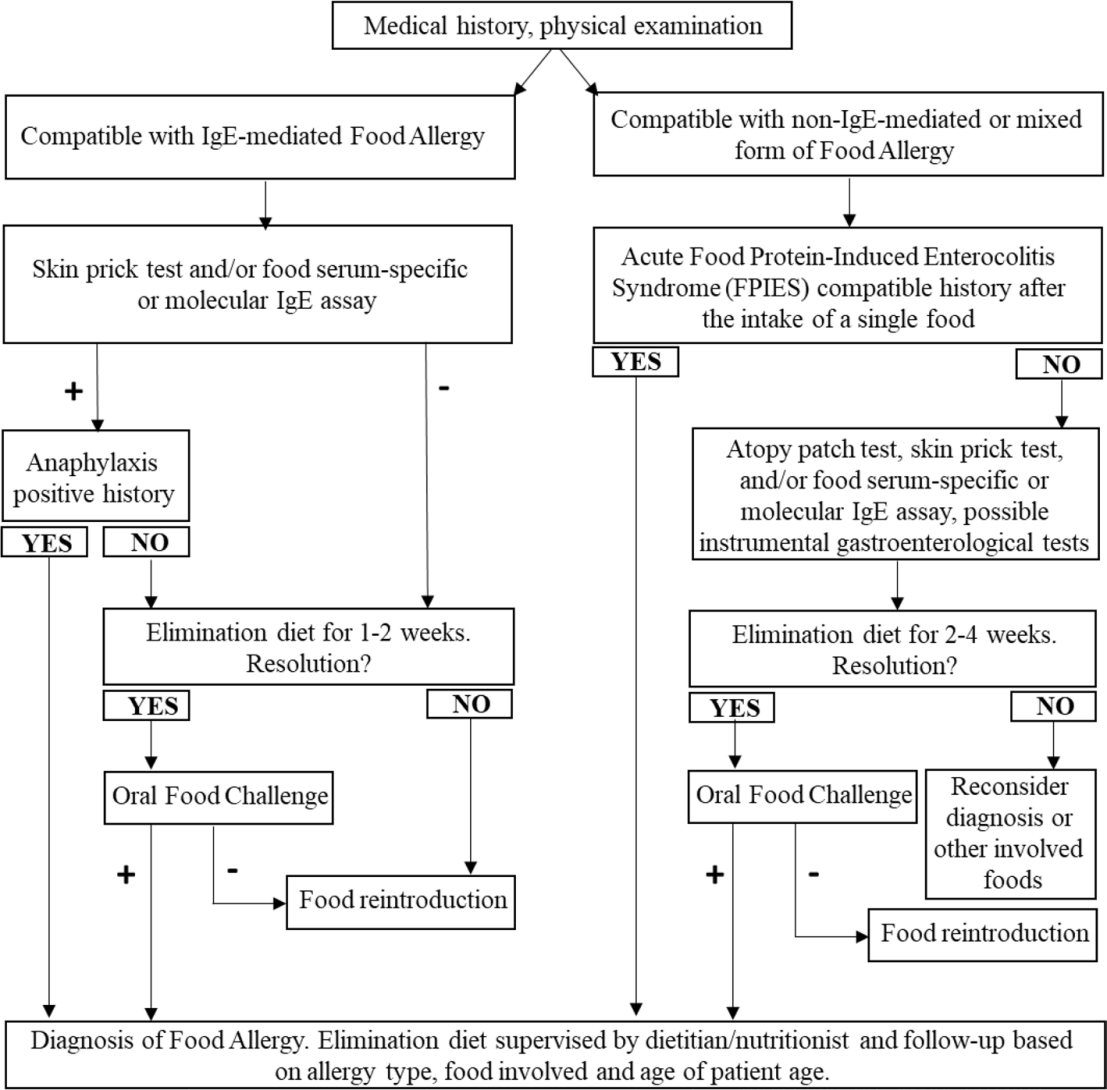
Diagnostic algorithm for the child with suspected food allergy. In case of Food-Dependent Exercise-Induced Anaphylaxis consider to perform allergy screening tests (skin prick test, food serum-specific IgE) and recommend abstention from physical exercise within 4–6 hours of suspect food and/or meal assumption. In the suspicion of eosinophilic pathologies of the gastrointestinal tract, the diagnosis is based on the response of the histological examination

A clinical picture characterized by gastrointestinal symptoms (diarrhea, blood and/or mucus in the stool, abdominal pain, retrosternal burning, regurgitation, vomiting, dysphagia, body growth failure, etc.), in addition to the screening allergy tests, may require the help of gastroenterological tests (endoscopy with histological examination, pH-impedance analysis, manometry, abdominal ultrasounds evaluation) to exclude and/or confirm mixed or non-IgE-gastrointestinal FA conditions [51]. In case of symptoms compatible with eosinophilic esophagitis (EoE) (vomiting, regurgitation, dysphagia, food bolus, retrosternal burning, etc.), in the presence or absence of peripheral eosinophilia (> 700 cells/mm^3^), it is necessary to carry out esophagogastroduodenoscopy (EGDS) by performing at least 4–6 esophageal biopsies including the proximal and distal parts of the esophagus. Diagnosis of EoE is based on the presence of esophageal dysfunction symptoms, eosinophilic infiltrate in the esophageal mucosa on histological examination (> 15 eosinophils in at least one high power field, HPF) which persist after at least 8 weeks with a proton pump inhibitor treatment (1–2 mg/kg/day) and when other causes of esophageal eosinophilia (gastroesophageal reflux disease, infectious esophagitis, achalasia, celiac disease and Crohn’s disease, connective tissue disorders, graft versus host disease, hypersensitivity to drugs and hypereosinophilic syndromes) are excluded. In case of symptoms characterized by abdominal pain, diarrhea, blood in the stool, in the presence or absence of peripheral eosinophilia, an endoscopy of the upper and/or lower intestinal tract with multiple biopsies is necessary. The finding of gastric (≥30/HPF to ≥5 HPF) and/or small intestine (duodenum: 50/HPF; jejunum: 2 × 26/HPF or 52/HPF; ileus: 2 × 28/HPF or 56/HPF) or colon (cecum and ascending colon: 2 × 50/HPF or 100/HPF; transverse and descending colon: 2 × 42/HPF or 84/HPF; rectus-sigma: 2 × 32/HPF or 64/HPF) eosinophilia, confirms the diagnosis of gastritis, gastroenteritis or eosinophilic colitis respectively [52], after excluding the secondary causes of intestinal eosinophilia (infectious enteritis, celiac disease, Crohn’s disease, connective tissue disorders, graft versus host disease, drug hypersensitivity, tumors of the blood compartment, X-linked syndrome of immunedysregulation-polyendocrinopathy-enteropathy (IPEX) and hypereosinophilic syndromes). Performing multiple biopsies of the esophageal and gastrointestinal tract (4–6 biopsies per segment) is essential for a correct diagnostic approach, as eosinophils are distributed in a focal way in enteritis; for this reason, the histological section can result falsely negative. The other non-IgE mediated forms of FA do not generally require instrumental tests, except in cases of persistent symptoms after 2–4 weeks of food elimination diet, to exclude other pathologies in differential diagnosis. Table 4 shows Diagnostic criteria for Eosinophilic Disorders of the Gastrointestinal Tract (***enter*** Table 4***here***). Figure 2 shows the Diagnostic Algorithm for the child with suspected FA [53].

**Table 4:** Diagnostic criteria for Eosinophilic Disorders of the Gastrointestinal Tract

	Symptoms	Number of eosinophils per field^a^
**Eosinophilic esophagitis**	Growth retardation, feeding difficulties, abdominal pain, non-specific symptoms of gastroesophageal reflux, recurrent vomiting, dysphagia and esophageal food impaction.	≥15/HPF
**Eosinophilic gastroenteritis**	*Mucosal form:* abdominal pain, vomiting, nausea, dyspepsia, diarrhea, malabsorption, protein-dispersing enteropathy with subsequent weight loss, anemia and hypoalbuminemia. *Muscle form:* reduced gastric motility, stiffening of the affected tract and possible intestinal obstruction. *Serum form:* irritation of the peritoneum with eosinophilic ascites, peritonitis and in the most severe cases intestinal perforation.	≥ 30/5 HPF at the gastric level ≥50/HPF at the duodenal level
**Eosinophilic colitis**	Abdominal pain, diarrhea and/or constipation, rectorrhagia, risk of acute complications such as volvulus and intussusception.	≥50/2 HPF or 100/HPF for cecum and ascending colon ≥42/2 HPF or 84/HPF for transverse colon and descending colon ≥32/2 HPF or 64/HPF for rectum and sigma

^a^4–6 biopsies/gastrointestinal segment are required

Abbreviation: *HPF* High-power field

### Therapeutic approach for pediatric food allergies

Currently, there is no drug therapy capable of preventing the allergic reaction after the ingestion of the offending foods. The standard and most effective FA treatment, once a FA is diagnosed, is the strict avoidance of the offending food or foods, as such and as a constituent of other foods. This strategy results simple when the food is not commonly consumed in the subject’s diet and does not have a high nutritional power. The food elimination diet becomes more complex if the offending food is very commonly consumed in the subject’s diet and has a high nutritional value. So, the certain identification of the offending food is an important goal to avoid life-threatening reactions and nutritional imbalances induced by inappropriate elimination diet. Sometimes the patient can assume the offending food involuntarily because as a constituent of other foods, therefore an adequate nutritional “counseling” plays a fundamental role in this setting. Prescribing a nutritionally adequate and medically safe diet is a focal point in the FA management. In the nutritional management of children with FA it is important to bestow the adequate amount of nutrients (carbohydrates, lipids, proteins, and micronutrients), make sure that there is adequate growth and that the diet allows as much as possible to practice a normal life of social relationship. So, the role of dieticians in the elaboration of dieto-therapeutic schemes is fundamental in the multidisciplinary management of FA pediatric patients.

Among pharmacological product for symptoms relief, antihistamines play an important role in case of symptoms of oral allergic syndrome or urticaria/angioedema. Oral corticosteroids are generally effective in treating both IgE-mediated and non-IgE-mediated forms and should always be available to the parents of child with FA along with antihistamines. In case of more severe reactions, the severity of the clinical picture requires treatment aiming the maintenance of vital functions. In case of anaphylaxis, the first-choice drug is adrenaline, which must be hand out by deep intramuscular route on the vastus lateral of the thigh at a dosage of 0.01 mg/kg of body weight, up to a dosage of 0.5 mg in the child, repeatable after 5 minutes in case of symptoms’ persistence or worsening. All patients with FA and with a history of anaphylaxis must be equipped with devices of pre-dosed adrenaline for self-injection to allow prompt intervention directly by the patient or family member (e.g., parents) in case of allergic reaction. Each patient and caregiver must be instructed on use of self-injectable adrenaline and on the procedures to be implement in case of anaphylaxis, alerting immediately the emergency room. The pharmacological products to use for the management of non-IgE-gastrointestinal FA or mixed forms are the proton pump inhibitors and/or topical or systemic steroids in case of eosinophilic pathologies of the gastrointestinal tract; rehydration, antiemetics (ondansetron) and cortisone in FPIES [54]. Elimination diet of offending food/s remains the only current strategy in other non-IgE-gastrointestinal FA forms.

Oral immunotherapy (OIT) is a potential treatment for IgE-mediated FA, in particular for milk and egg, in order to increase the oral tolerance threshold and/or induce/accelerate the food oral tolerance process; this procedure can be conducted after the obtainment of parents’ children informed consent in highly specialized centers.

Oral tolerance to offending food is naturally reached with growth in over 90% of children affected by cow’s milk protein and eggs allergies; while IgE-mediated FA to fish, mollusks, crustaceans, and nuts resolves in less than 20% of pediatric patients and tends to persist throughout life in most of cases [1].

### Diagnostic and therapeutic approach for pediatric food intolerances

Carbohydrate intolerances (CI) are the most common form of FI. Symptoms are mainly due to the lack of enzymes, transporters, or the overload of transport systems in the intestinal epithelium. The non-absorbed carbohydrates recall fluids by osmosis in the intestinal lumen causing osmotic diarrhea and intestinal bacterial fermentation with gas production and consequent distension and abdominal pain, flatulence, nausea and increased intestinal motility. Extra-intestinal symptoms, such as headache, dizziness, memory disturbances and lethargy, may rarely occur. In some case the symptoms can occur in the first stage of life with very severe gastrointestinal picture and early diagnosis even with genetic tests is needed.

#### Glucose-galactose malabsorption

It is a very early onset food intolerance characterized by diarrhea and severe dehydration starting from the neonatal period. A modest glycosuria is also present, while fructose absorption is normal. Glucose-galactose malabsorption is due to a mutation in the SLC5A1 gene, which encodes the glucose-sodium co-transporter SGTL1. Transmission is autosomal recessive. A molecular diagnosis of the condition by specialized centers is possible. An elimination diet of galactose and glucose quickly resolves the symptoms [55].

#### Lactose intolerance

Lactose intolerance is the most frequent form of CI in children and is characterized to the inability to digest lactose due to lack or deficiency of the lactase enzyme responsible for the digestion of lactose into glucose and galactose.

Based on the etiology, lactose intolerance can be classified into three main forms:

- *Congenital lactase deficiency*: a rare autosomal recessive disease in which enzyme activity is absent or reduced from birth.
- *Secondary lactase deficiency*: a common consequence of mucosal diseases such as bacterial proliferation in the small intestine, infections, celiac disease, Crohn’s disease or radiation enteritis.
- *Adult-type hypolactasia (also known as lactase non-persistence)*: an autosomal recessive condition resulting from a mutation in the product of lactase gene, responsible for the reduced synthesis of the precursor protein.

Medical history and lactose breath test are the main tools for the diagnosis of lactose intolerance. The management consists in the exclusion from the diet of foods containing lactose (see Table 5, column concerning lactose). In adult-type hypolactasia, foods containing lactose are generally excluded for 2–4 weeks, which is the time useful for symptoms solving. Subsequently, a gradual reintroduction of these foods is carried out until the tolerated dose is reached.

**Table 5:** Main foods containing FODMAPs

Lactose	Fructose	Fructans	Galactans	Polyols
Milk Butter Sour cream Condensed milk Ricotta Creamy cheeses Spreadable cheeses Mozzarella Ice cream Yogurt	Fruits: apricots, avocado, persimmons, cherries, watermelon, dates, figs, mangoes, apples, papaya, pears, peaches, plums Marmalade Fruit juices Dried and canned fruit Honey and molasses	Vegetables: garlic, asparagus, beets, broccoli, artichokes, Brussels sprouts, cauliflower, cabbage, onions, green beans, fennel, mushrooms, leeks Cereals: wheat, spelled, barley, kamut, rye	Legumes: beans, chickpeas, peas, lentils	Fruits: apples, apricots, cherries, peaches, pears, plums, watermelon Vegetables: cauliflower, mushrooms Sweeteners: sorbitol, mannitol, maltitol, xylitol

Abbreviation: *FODMAPs* Fermentable oligosaccharides, disaccharides, monosaccharides and polyols

In secondary lactase deficiency, the lactose elimination diet is required for a limited time. Literature data suggest that adults and adolescents with lactose intolerance can take up to 12 g of lactose in a single dose (corresponding to a cup of milk) in absence of symptoms or with minor symptoms. Subjects with lactose intolerance may be at risk of reduced calcium intake and supplementation may be required in accordance with current recommendations.

#### Sucrose-isomaltose malabsorption

It is secondary to a congenital sucrase-isomaltase deficiency (CSID) characterized by an abnormal absorption of oligosaccharides and disaccharides. Breastfed infants or infants fed with formula containing exclusively lactose are asymptomatic. Symptoms such as watery osmotic diarrhea, abdominal distension and vomiting occur when sucrose or starch dextrins are introduced into the diet. Symptom severity can cause stunting, dehydration, and malnutrition. The CSID is inherited as an autosomal recessive trait and is due to mutations of the sucrase-isomaltase complex (SI) necessary for the digestion of sucrose and starch into monosaccharides on the enterocyte apical membrane. The diagnosis is based on the presence of osmotic diarrhea and is confirmed by positive sucrose breath test. In specialized centers a molecular diagnosis of the condition could be also performed. The management consists of a low sucrose and starch diet. The sucrase-isomaltase prognosis is good, because the starch intolerance resolves during the first years of life and sucrose intolerance usually improves with age [56].

#### Fructose malabsorption

Fructose is a six-carbon monosaccharide naturally present in fruits, vegetables, and honey. High fructose syrup (HFC) can be obtained in food industry through the enzymatic hydrolysis of corn starch, and it could use as a sweetener in soft drinks, candies, and fruit juices.

Fructose malabsorption should not be confused with hereditary fructose intolerance (HFI), in which the lack of aldolase B enzyme leads to an accumulation of fructose-1-phosphate in the liver, kidney and intestines, causing hypoglycemia, nausea, swelling, pain abdominal, diarrhea and vomiting. The specific mechanism responsible for fructose malabsorption is not yet known, but this disorder may be secondary to intestinal damage (e.g., induced by diseases such as celiac disease).

The diagnosis of fructose malabsorption can be performed through the hydrogen breath test after oral fructose load, although some studies have shown a high percentage of false negative results of this test. The fructose malabsorption management is based on a daily dietary intake of fructose less than 10 g and on the exclusion from the diet of alcoholic beverages [55].

#### Sorbitol intolerance

Sorbitol is a sugar naturally present in fruit and fruit juices and it is also used in commercial products such as drugs, sweets, dietetic foods and chewing gum. The hydrogen breath test after oral sorbitol load is effective in identifying this condition. The main therapeutic approach is characterized by a reduced content of sorbitol in the diet [55].

#### FODMAPs intolerance

Fermentable monosaccharides, disaccharides, oligosaccharides, and polyols (FODMAPs) are a group of short-chain carbohydrates that are poorly absorbed in the intestine. These highly osmotic substances are fermented by the intestinal bacteria and can evoke the onset of gastrointestinal symptoms by distension of the lumen or by direct action on the intestine through not well-defined mechanisms. The therapeutic approach of FODMAPs intolerance is based on an exclusion diet of foods containing FODMAPs (Table 5**- enter here at the end of sentence**). Considering the large number of foods containing FODMAPs and that the FODMAPs tolerance threshold could be different between subjects, the exclusion diet must be carefully tailored by an experienced nutritionist based on clinical history and the result of the hydrogen breath test. FODMAPs-free diet is usually recommended for 4–6 weeks. After this period, patients are invited to try to reintroduce one or more FODMAPs containing foods, to ascertain their intolerance and/or to assay the FODMAPs tolerance threshold [55].

#### Non-celiac gluten sensitivity

Non-celiac gluten sensitivity (NCGS) is a syndrome characterized by persistent gastrointestinal and/or extraintestinal symptoms related to the ingestion of gluten-containing foods that resolve after gluten-free diet, in subjects that are not affected by either celiac disease or wheat allergy. Symptoms generally appear within hours or days after the ingestion of gluten-containing foods and disappear just as quickly with the start of a gluten-free diet [50]. In pediatric age NCGS is rare and mainly affects males. The most frequent symptoms are abdominal pain and chronic diarrhea; but vomiting, constipation, bloating, poor growth, asthenia, headache may be also present. Extraintestinal symptoms (chronic fatigue, joint and muscle pain, headache, depression, “foggy mind”, eczema, anemia) are mostly reported in adulthood. The pathogenetic mechanism is unknown. NCGS is associated in one third of patients with food intolerances to other foods (especially lactose intolerance), in 20% IgE-mediated inhalant allergies subjects. The diagnosis is mainly clinical and requires the exclusion of celiac disease and wheat allergy diagnosis. The diagnostic gold standard is the clinical benefit to the gluten-free diet, followed by a double-blind placebo-controlled gluten challenge. In the period prior to the gluten-free diet, the subject must lead a diet containing gluten for at least 6 weeks; the subsequent gluten-free diet must be strict to avoid any contamination and last at least 6 weeks.

The daily dose of gluten to take in the double-blind placebo-controlled gluten challenge is approximately 8 g (with 0.3 g of amylase-trypsin inhibitors, ATI) in a vehicle FODMAPs free. The placebo must be completely gluten-free. The first test phase should last at least 7 days, followed by a wash out period of 7 days, and a second test phase of at least 7 days.

The NCGS management is based on gluten-free diet; there is no indication to eliminate possible gluten contamination in foods, as is instead necessary in celiac disease [57].

### Toward an integrated diagnostic-therapeutic pathway for the pediatric patient with FAI

As depicted in Fig. 1, the healthcare professionals should make their expertise available to the patients in a circular continuum of activities. The first step is the appropriate recognition of the different forms of FAIs based on the correct evaluation of the anamnestic and clinical features of the patient. The onset of symptoms, with varying degrees of severity, can be acute, chronic, episodic, or recurrent. The patient with severe acute FAI-induced symptoms is commonly observed by the physician operating in the ED. At the ED the patient should receive rapid recognition of the disorder and adequate treatment to obtain a faster symptoms resolution. After full stabilization of the symptoms, the physician operating at the ED can refer the patient to the FP or to the tertiary center for the diagnosis and treatment of FAI (protected outpatient pathway). In any case, at discharge, all patients should receive clear indication regarding home therapy along with indications on the elimination diet pending the subsequent evaluation planned at the tertiary center. If the patient is primarily observed by the FP, he should carefully assess the anamnestic and clinical features of the child and the possible chronological relationship between ingestion of the food and the occurrence of symptoms. Concomitantly, the FP should treat any symptom that may still be present. In case of a well-founded suspicion of a food/symptom cause-effect relationship, the FP should request a specialist assessment by the tertiary center for pediatric FAI according to the priority criteria. Alternatively, the FP can also only take note of the clinical documentation relating to the access to the ED and in this case should refer the patient to the tertiary center. Particular attention should be paid to subjects with acute symptoms that involve multiple organs in rapid succession: it can be an anaphylactic event that must be treated immediately at the ED.

### Activities, skills, roles, and responsibilities of healthcare professionals

#### General practitioner/family pediatrician

- to raise the diagnostic suspicion of FAI, eventually based on the result of SPT and/or serum specific IgE
- to prescribe symptom therapy (antihistamines and/or steroids) in case of “in progress” symptoms
- to provide initial indications on elimination diet and the management of any symptom exacerbations
- to refer the patient to the tertiary center for pediatric FAI

#### Emergency department physician

- to raise the diagnostic suspicion of FAI
- to prescribe symptom therapy (antihistamines and/or steroids and/or adrenaline) if it is required
- to measure serum tryptase if the symptoms have arisen for less than 4 hours in case of anaphylaxis
- to provide initial diet indications and symptoms exacerbations management
- to prescribe or equip the patient with self-injectable adrenaline if necessary
- to refer the patient to the tertiary center for pediatric FAI

#### Tertiary center physician for pediatric FAI

- to frame the clinical case and coordinate the implementation of the necessary and appropriate diagnostic procedures: OFC and/or laboratory-instrumental analysis (SPT, prick by prick, APT, breath test, endoscopy with histology, pH-impedance analysis, imaging, non-invasive tests of intestinal function, etc.) to perform a conclusive diagnosis of FAI
- to plan a follow-up program to provide guidance on management, prognosis, therapy, and prevention of new events and, if necessary, to prescribe or equip the patient with self-injectable adrenaline
- to provide a “nutritional counseling” for an adequate elimination diet

#### Pediatric nurse

- to perform a correct triage when the patient is observed at the ED
- to monitor the patient carefully looking for any symptoms of local or systemic reactions, to administer first aid drugs in case of adverse reaction, to record the procedure, the patient tolerance, and each detection during the test
- to know how to perform allergy skin tests
- to assist and collaborate with the physician in carrying out allergy tests (SPT, prick by prick, APT, OFC)
- to assist the operator in carrying out instrumental gastroenterological diagnostic tests (breath test, endoscopy, pH-impedance analysis)

#### Dietitian/nutritionist

- to assess the nutritional status and dietary habits (e.g., 3- or 7-days food diary)
- to prescribe a nutritionally adequate diet eliminating the offending food/s and proposing optimal alternatives to achieve a full adherence to the elimination diet
- to assess the patient with periodic follow-up

## Conclusion

The DTCP is devoted to all healthcare professional approaching pediatric subjects with suspected FAIs. The DTCP will facilitate the early recognition and the management of FAIs in the pediatric age. The appropriate application of this DTCP will reduce not only delays and diagnostic errors, but also health risks for children affected by FAIs facilitating a rationale approach to these conditions with a reduction of socioeconomic costs for the families and the health care system.

## Data Availability

No datasets were generated or analyzed during the current paper.

## Abbreviations

AGA: American Gastroenterology Association
ALPHA-GAL: Galactose-alpha-1,3-galactose
APT: Atopy Patch Test
ATI: Amylase-trypsin inhibitors
CCD: Cross-reactive carbohydrate determinants
CI: CARBOHYDRATE intolerances
CMA: Cow’s milk allergy
CRD: Component-resolved diagnostic
CSID: Congenital sucrase-isomaltase deficiency
DTCP: Diagnostic Therapeutic Care Pathway
EAACI: European Academy of Allergy and Clinical Immunology
ED: Emergency Department
EGDS: Esophagogastroduodenoscopy
EoE: Eosinophilic esophagitis
FA: Food allergies
FAIs: Food allergies and intolerances
FI: Food intolerances
FODMAPs: Oligo-, Di-, Monosaccharides and Fermentable Polyols
FP: Family Pediatrician
FPIES: Food Protein-Induced Enterocolitis Syndrome
GRP: Gibberellin-regulated protein
HFC: High fructose syrup
HFI: Hereditary fructose intolerance
HPF: High power field
IPEX: X-linked syndrome of immunedysregulation-polyendocrinopathy-enteropathy
ISTAT: Italian National Institute of Statistics
LTP: Lipid Transfer Proteins
NGCS: Non-celiac gluten sensitivity
OAS: Oral allergic syndrome
OFC: Oral Food Challenge
OIT: Oral immunotherapy
PR-10: Pathogenesis Related Proteins-10
SI: Sucrase-isomaltase
SIAIP: Italian Society for Pediatric Allergy and Immunology
SIGENP: Italian Society for Pediatric Gastroenterology Hepatology and Nutrition
SPT: Skin prick tests
